# Comparison of Four Lymph Node Staging Systems in Gastric Adenocarcinoma after Neoadjuvant Therapy – A Population-Based Study

**DOI:** 10.3389/fsurg.2022.918198

**Published:** 2022-05-27

**Authors:** Hongkun Lai, Jiabin Zheng, Yong Li

**Affiliations:** ^1^ The Second School of Clinical Medicine, Southern Medical University, Guangzhou, China; ^2^ Department of Gastrointestinal Surgery, Guangdong Provincial People’s Hospital; Guangdong Academy of Medical Sciences, Guangzhou, China

**Keywords:** neoadjuvant therapy, prognosis, lymph node classification, gastric adenocarcinoma, lymph node ratio, positive logarithm ratio of lymph node, negative lymph node

## Abstract

**Introduction:**

Neoadjuvant treatment leads in a reduction in positive lymph nodes and examined lymph nodes (ELN), which may affect assessment of lymph node staging and postoperative treatment. We aimed to compare the staging systems of lymph node ratio (LNR), the positive logarithm ratio of lymph nodes (LODDS), negative lymph nodes (NLN), and the 8th AJCC ypN stage for patients with gastric adenocarcinoma after neoadjuvant therapy.

**Materials and Methods:**

Data was collected from the Surveillance, Epidemiology, and End Results database and 1,551 patients with gastric adenocarcinoma who underwent neoadjuvant therapy and radical surgery were enrolled. Harrell’s concordance index, the Receiver Operative Curve, the likelihood ratio test, and the Akaike information criterion were used to compare the predictive abilities of the different staging systems.

**Results:**

Among the 1,551 patients, 689 (44.4%) had ELN < 16 and node-negative patients accounted for 395 (25.5%). When regarded as the categorical variable, LNR had better discrimination power, higher homogeneity, and better model fitness for CSS and OS compared to other stage systems, regardless of the status of ELN. When regarded as the continuos variable, LODDS outperformed others for CSS. Furthermore, the NLN staging system performed superior to others in node-negative patients.

**Conclusions:**

LNR had a better predictive performance than ypN, LODDS and NLN staging systems regardless of the status of ELN when regarded as the categorical variable, whereas LOODS became the better predictive factor for CSS when regarded as the continuos variable. In node-negative patients, NLN might be a feasible option for evaluating prognosis. A combination of LNR and NLN should be considered as user-friendly method in the clinical prognostic assessment.

## Introduction

At present, neoadjuvant therapies have become the standard for the management of patients with locally advanced gastric adenocarcinoma in western countries, based on the results of randomized controlled trials [1–6]. Compared with patients with initial surgical resection, fewer examined lymph nodes (ELN) and positive lymph nodes (PLN) were harvested in patients receiving neoadjuvant therapy (NAT) [7], particularly in patients treated with neoadjuvant radiation [8]. As a result, neoadjuvant therapy increased the number of node-negative patients as well as those with insufficient ELN [7], and it may not accurately assess lymph node status following NAT. However, the lymph node (LN) status in the AJCC ypTNM stage system is still based on the number of PLN and it is recommended to harvest at least 16 LN to reduce the stage migration. It was proved that ELN in node-negative patients after NAT was an independent prognostic factor and the patients had better survival with ELN increased, suggesting that patients with insufficient ELN may be underestimated and when ELN increased, the patients may upstage to ypN1 or ypN2 [9]. Previous research suggested that LN status could aid in determining postoperative treatment in patients with pathological complete response [10–12]. Currently, the 8th edition AJCC ypTNM stage system is the most widely used to predict the prognosis of patients receiving NAT. Lymph node ratio (LNR) has been shown to be an alternative prognostic factor in a variety of cancers, and it has been proposed that it may reduce the N-stage migration effect [13–16]. However, in several studies, it was suggested that for patients with no metastatic lymph node, LNR does not outperform the ypN stage system [8, 17]. Moreover, LODDS is the logarithm of the ratio between PLN and negative lymph nodes (NLN) and has been proven to reduce the stage migration in many cancers, including pancreatic cancer and gastric adenocarcinoma [8, 18, 19]. Some studies have shown that NLN is also an alternative stage for a predictive prognosis for gastric adenocarcinoma and breast cancer [20, 21]. However, most previous studies have focused on patients who have not received preoperative treatment [22–26]. Whether the conclusions from these studies could be extended to patients after NAT remains unclear. In this study, we aimed to identify the best staging system among ypN, LNR, LODDS, and NLN for the prediction of prognosis in patients with gastric adenocarcinoma after NAT.

## Materials and Methods

**Figure 1:**
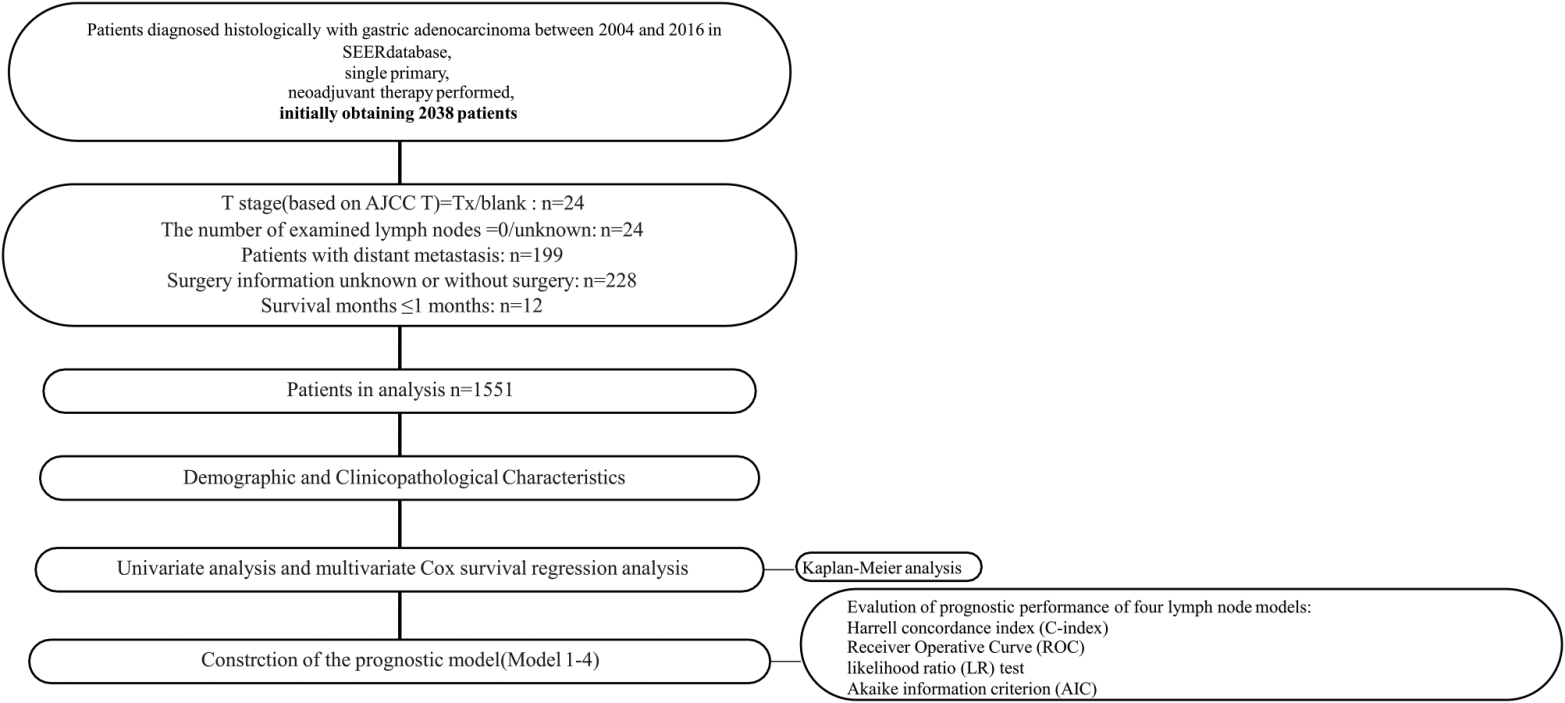
Research flowchart.

All procedures involving human participants in this study comply with the Declaration of Helsinki (revised in 2013). Due to the retrospective nature of the study, the requirement for informed consent was abandoned. SEER*Stat software (Version 8.3.2) was used to collect data from the Surveillance, Epidemiology, and End Result (SEER) database (1973–2016). 1,551 patients with primary gastric adenocarcinoma who received NAT and surgical resection were enrolled (Figure 1).

### Patients

Data collected included patient demographic information, clinicopathological characteristics, and survival. The inclusion criteria were as follows: (1) Age ≥18 years. (2) There is no history of the malignant tumor. (3) The histopathological diagnosis is gastric adenocarcinoma (5) Completed follow-up and the postoperative survival time ≥2 months. (6) Neoadjuvant therapy is performed before the operation. The primary outcomes were cancer-specific survival (CSS) and overall survival (OS). CSS is defined as the time from diagnosis to cancer-specific death, and OS is defined as the interval between the date of diagnosis and the date of all-cause death.

### Tumor Stage and LN Classifications

We defined the tumor stage using the 8th edition AJCC ypTNM staging system and conducted an appropriate conversion in patients whose tumor stage was presented with old TNM editions. 8th AJCC ypN stage (ypN0: no PLN, ypN1: 1–2 PLN, ypN2: 3–6 PLN, ypN3: PLN ≥ 7) is the current standard for the lymph node stage of gastric adenocarcinoma. LNR is the ratio of the number of PLN to the number of ELN (LNR = PLN/ELN). [27] Use Xtile software (Yale University, New Haven, CT, USA) to obtain the best cut-off value of three alternative staging systems (LNR, LOODS, NLN) and divide the patients into 4 groups,respectively [28]. LNR:LNR1:0; LNR2: 0.01 ≤ LNR ≤ 0.14; LNR3: 0.14 < LNR ≤ 0.37, LNR4: LNR >0.37. LODDS is the logarithm of (PLN + 0.5)/(NLN + 0.5). The patients were designated according to cut-off values of −2.75, −1.61 and −0.51: LODDS1: LODDS ≤ −2.75;LODDS2: −2.75 < LODDS ≤ −1.61; LODDS3: −1.61 < LODDS ≤ −0.51; LODDS4:LODDS > −0.51. NLN is the value of (ELN-PLN).NLN was designed into NLN1: NLN1 (NLN > 21), NLN2 (13 < NLN ≤ 21), NLN3 (8 < NLN ≤ 13), NLN4 (NLN ≤ 7). We analyzed the four staging systems as categorical and continuous variables, respectively.

### Statistical Analysis

R software (version 3.61) and SPSS 25.0 (IBM SPSS Inc.) were used for statistical analysis. The baseline characteristics of patients were statistically analyzed by quantitative values and median interquartile differences (IQRs). Baseline factors related to CSS and OS were evaluated by univariate and multivariate Cox survival Regression Analysis. Multivariate analysis models included the covariates T stage, age, histological grade, tumor location, histological type, and each staging system, that is, ypN stage (Model1), LNR (Model2), LODDS (Model3), NLN (Model4). The Kaplan-Meier method was used to estimate CSS and OS, and the Log-rank test was used to verify the survival difference of each subgroup. The performance of the prognostic system has been proved to be evaluated by the discriminatory power (the survival of patients in different stages of each system is more different) and homogeneity (the survival of patients of the same category is less different in each system) [29–31]. Harrell’s c index (C-index) and the Receiver Operative Curve (ROC) were performed to compare the discriminatory power among different systems. Model fit was evaluated using the Akaike information criterion (AIC) [31]. The likelihood ratio test (LR test) was calculated using Cox regression to measure homogeneity; a higher likelihood ratio test indicates better homogeneity. An internal validation procedure using 2,000 bootstraps was applied to the calculation of the C-index and Confidence Intervals. All tests are two-sided tests, and *p* < 0.05 is considered statistically significant.

## Results

### Demographic and Clinicopathological Characteristics

A total of 1,551 patients were eligible for inclusion in this study. The patients’ characteristics are summarized in Table 1. 395 (25.5%) were female and 1,156 (74.5%) male. 589(38.0%)were more than 65 years old. After NAT, most patients were found to have poorly or undifferentiated adenocarcinoma. Patients had a median of 17 lymph nodes examined. Among them, ELN ≥ 16 was 862 (55.6%) and ELN < 16 was 689 (44.4%), and node-negative patients were 395 (25.5%), and the median number of lymph node metastases was 2 (range from 0 to 47). The overall survival time have been described in section “Survival Analysis” below.

**Table 1:** Baseline characteristics and univariate analysis for CSS and OS in gastric adenocarcinoma patients after NAT.

Characteristic	*N* (%/IQR)	CSS		OS	
	1,551	Hazard ratio (95% CI)	*p-*value	Hazard ratio (95% CI)	*p-*value
Age					
≤65	962 (62.0)	1.00			1
>65	589 (38.0)	1.09 (0.95–1.25)	0.24	1.16 (1.02–1.32)	0.03
Race					
White	1,198 (77.2)	1.00		1	
Black	147 (9.5)	0.97 (0.77–1.22)	0.78	0.94 (0.76–1.18)	0.60
Other	206 (13.3)	0.79 (0.64–0.97)	0.03	0.83 (0.69–1.02)	0.07
Sex					
Female	395 (25.5)	1.00		1	
Male	1,156 (74.5)	0.97 (0.84–1.13)	0.71	1.02 (0.88–1.18)	0.76
Location					
Upper	928 (59.8)	1.00		1	
Middle	259 (16.7)	0.78 (0.64–0.95)	≤0.01	0.82 (0.69–0.99)	0.04
Lower	188 (12.1)	0.87 (0.7–1.08)	0.21	0.87 (0.71–1.07)	0.20
Overlapping	106 (6.8)	1.22 (0.95–1.58)	0.12	1.18 (0.92–1.52)	0.19
NOS	70 (4.5)	1.46 (1.08–1.96)	≤0.01	1.51 (1.14–2)	≤0.01
Histologic type					
Adenocarcinoma	1,225 (79.0)	1.00		1	
Signet ring cell carcinoma	326 (21.0)	1.38 (1.18–1.61)	≤0.01	1.32 (1.14–1.53)	≤0.01
Grade					
G1/G2	399 (25.7)	1.00		1	
G3/G4	1,053 (67.9)	1.42 (1.21–1.67)	≤0.01	1.33 (1.15–1.55)	≤0.01
Unknown	99 (6.4)	1.20 (0.88–1.62)	0.24	1.1 (0.82–1.47)	0.51
Tumor size					
≤2 cm	204 (13.2)	1.00		1	
>2 cm, ≤5 cm	590 (38.2)	1.25 (1–1.56)	0.05	1.25 (1.01–1.54)	0.04
>5 cm	476 (30.8)	1.46 (1.16–1.83)	≤0.01	1.43 (1.16–1.78)	≤0.01
Linitis plastica	27 (1.7)	1.73 (1.06–2.82)	0.03	1.71 (1.07–2.72)	0.02
Uknown	249 (16.1)	1.18 (0.92–1.53)	0.20	1.18 (0.92–1.5)	0.19
ypT category					
ypT1	124 (8.0)	1.00		1	
ypT2	159 (10.3)	1.62 (1.11–2.39)	≤0.01	1.45 (1.03–2.05)	0.03
ypT3	652 (42.0)	2.28 (1.65–3.15)	≤0.01	1.94 (1.46–2.59)	≤0.01
ypT4a	506 (32.6)	2.80 (2.02–3.88)	≤0.01	2.38 (1.79–3.18)	≤0.01
ypT4b	110 (7.1)	3.41 (2.33–5)	≤0.01	2.9 (2.06–4.1)	≤0.01
Positive lymph nodes	2.00 [0.00, 6.00]	0.99 (0.99–1)	≤0.01	1.06 (1.05–1.07)	≤0.01
Examined lymph nodes	17.00 [11.00, 25.00]	1.07 (1.06–1.08)	≤0.01	0.99 (0.99–1)	≤0.01
ypN category					
ypN0	395 (25.5)	1.00		1	
ypN1	413 (26.6)	1.60 (1.31–1.97)	≤0.01	1.52 (1.26–1.84)	≤0.01
ypN2	404 (26.0)	2.11 (1.73–2.58)	≤0.01	1.89 (1.57–2.28)	≤0.01
ypN3	339 (21.9)	3.40 (2.77–4.17)	≤0.01	3.08 (2.55–3.73)	≤0.01
ELN (categorical)					
ELN ≥ 16	862 (55.6)	1.00		1	
ELN < 16	689 (44.4)	1.17 (1.02–1.33)	0.02	1.15 (1.01–1.3)	0.03
Lymph node ratio	0.14 [0.00, 0.37]	6.85 (5.44–8.64)	≤0.01	6.49 (5.2–8.1)	≤0.01
LNR category					
LNR1	395 (25.5)	1.00		1	
LNR2	394 (25.4)	1.36 (1.1–1.68)	≤0.01	1.28 (1.05–1.55)	0.02
LNR3	376 (24.2)	2.11 (1.72–2.59)	≤0.01	1.91 (1.58–2.31)	≤0.01
LNR4	386 (24.9)	3.81 (3.13–4.65)	≤0.01	3.48 (2.9–4.18)	≤0.01
LODDS	−1.61 [−2.75, −0.51]	1.40 (1.34–1.46)	≤0.01	1.38 (1.32–1.43)	≤0.01
LODDS category					
LODDS1	394 (25.4)	1.00		1	
LODDS2	389 (25.1)	1.45 (1.17–1.79)	≤0.01	1.32 (1.08–1.61)	≤0.01
LODDS3	384 (24.8)	2.20 (1.79–2.7)	≤0.01	1.97 (1.62–2.38)	≤0.01
LODDS4	384 (24.8)	3.94 (3.22–4.82)	≤0.01	3.55 (2.95–4.28)	≤0.01
NLN	13.00 [7.00, 21.00]	0.97 (0.96–0.98)	≤0.01	0.97 (0.97–0.98)	≤0.01
NLN category					
NLN1	359 (23.1)	1		1	
NLN2	383 (24.7)	1.32 (1.07–1.64)	≤0.01	1.31 (1.07–1.6)	≤0.01
NLN3	403 (26.0)	1.8 (1.47–2.21)	≤0.01	1.72 (1.42–2.09)	≤0.01
NLN4	406 (26.2)	2.29 (1.87–2.8)	≤0.01	2.19 (1.82–2.65)	≤0.01
Chemotherapy					
None	14 (0.9)	1.00		1	
Yes	1,537 (99.1)	1.51 (0.68–3.37)	0.31	1.49 (0.71–3.15)	0.29
Radiotherapy					
None	660 (42.6)	1.00		1.00	
Radiation prior to surgery	668 (43.1)	1.18 (1.01–1.36)	0.03	1.20 (1.05–1.39)	≤0.01
Radiation after surgery	201 (13.0)	1.22 (1.00–1.50)	0.05	1.20 (0.99–1.46)	0.07
Radiation before and after surgery	22 (1.4)	1.04 (1.57–1.90)	0.90	1.12 (0.64–1.94)	0.70

*OS, overall survival; CSS, cancer specific survival; N, number; IQR, interquartile range; HR, hazard ratio; CI, confidence interval; ELNs, Examined lymph nodes; PLN, number of positive lymph nodes; LNR, lymph node ratio; LODDS, log odds of positive lymph node; NLN, negative lymph node.*

### Univariate Analysis and Multivariate Analysis

As shown in Table 1, grade, histologic type, tumor size, ypT category, ELN, ypN stage, LODDS, LNR, NLN were significantly correlated with CSS, and age, grade, histologic type, tumor size, four lymph node staging schemes, ELN were significantly correlated with OS. Based on the univariate analysis and clinical practice, ypN (Model1), LNR (Model2), LODDS (Model3), and NLN (Model4) were respectively included in different multivariate Cox regression models for CSS and OS. As shown in Tables 2, 3, in the multivariate Cox regression models, four lymph node staging schemes (ypN, LNR, LODDS and NLN) were independent prognostic factors for CSS and OS.

**Table 2:** Multivariate Cox regression analysis of prognostic predictors for OS in gastric adenocarcinoma patients after NAT.

Characteristic	Model 1 (ypN)		Model 2 (LNR)		Model 3 (LODDS)		Model 4 (NLN)	
OS	Hazard ratio (95% CI)	*p-*value	Hazard ratio (95% CI)	*p-*value	Hazard ratio (95% CI)	*p* value	Hazard ratio (95% CI)	*p*-value
Age								
≤65	1.00		1.00		1.00		1.00	
>65	1.21 (1.06–1.38)	≤0.01	1.22 (1.07–1.39)	≤0.01	1.22 (1.07–1.39)	≤0.01	1.21 (1.06–1.38)	≤0.01
Histologic type								
Adenocarcinoma	1.00		1.00		1.00		1.00	
Signet ring cell carcinoma	1.23 (1.04–1.44)	≤0.01	1.21 (1.03–1.43)	0.02	1.21 (1.03–1.42)	0.02	1.20 (1.02–1.41)	0.03
Grade								
G1/G2	1.00		1.00		1.00		1.00	
G3/G4	1.12 (0.95–1.32)	0.18	1.14 (0.97–1.34)	0.12	1.15 (0.97–1.35)	0.10	1.21 (1.03–1.42)	0.02
Unknown	0.91 (0.68–1.23)	0.55	0.91 (0.68–1.23)	0.55	0.92 (0.68–1.23)	0.57	1.00 (0.74–1.34)	0.98
Location								
Upper	1.00		1.00		1.00		1.00	
Middle	0.76 (0.63–0.92)	≤0.01	0.76 (0.63–0.92)	≤0.01	0.76 (0.63–0.92)	≤0.01	0.80 (0.66–0.97)	0.02
Lower	0.77 (0.63–0.96)	0.02	0.77 (0.62–0.95)	≤0.01	0.77 (0.62–0.95)	≤0.01	0.79 (0.64–0.97)	0.02
Overlapping	0.93 (0.72–1.22)	0.62	0.94 (0.72–1.22)	0.63	0.94 (0.72–1.23)	0.64	0.99 (0.76–1.3)	0.97
NOS	1.06 (0.78–1.43)	0.72	1.03 (0.77–1.4)	0.82	1.04 (0.77–1.4)	0.82	1.17 (0.86–1.58)	0.31
Tumor size								
≤2 cm	1.00		1.00		1.00		1.00	
>2 cm, ≤5 cm	1.06 (0.86–1.32)	0.57	1.10 (0.88–1.36)	0.40	1.11 (0.89–1.37)	0.36	1.19 (0.96–1.47)	0.12
>5 cm	1.04 (0.83–1.31)	0.74	1.06 (0.85–1.34)	0.60	1.08 (0.86–1.35)	0.53	1.24 (0.99–1.56)	0.06
Linitis plastica	1.17 (0.72–1.92)	0.52	1.16 (0.71–1.9)	0.55	1.17 (0.72–1.92)	0.52	1.08 (0.66–1.77)	0.75
Uknown	1.00 (0.78–1.28)	0.99	1.03 (0.81–1.32)	0.81	1.03 (0.8–1.32)	0.82	1.05 (0.82–1.35)	0.69
ypT category								
ypT1	1.00		1.00		1.00		1.00	
ypT2	1.29 (0.91–1.83)	0.15	1.25 (0.88–1.77)	0.21	1.26 (0.89–1.79)	0.19	1.31 (0.92–1.85)	0.13
ypT3	1.49 (1.11–2)	≤0.01	1.41 (1.05–1.9)	0.02	1.41 (1.05–1.9)	0.02	1.64 (1.23–2.2)	≤0.01
ypT4a	1.65 (1.22–2.23)	≤0.01	1.57 (1.16–2.12)	≤0.01	1.56 (1.15–2.11)	≤0.01	1.81 (1.34–2.44)	≤0.01
ypT4b	1.89 (1.31–2.72)	≤0.01	1.79 (1.25–2.59)	≤0.01	1.80 (1.25–2.59)	≤0.01	2.15 (1.5–3.08)	≤0.01
ypN category								
ypN0	1.00							
ypN1	1.38 (1.14–1.68)	≤0.01						
ypN2	1.81 (1.49–2.21)	≤0.01						
ypN3	2.95 (2.39–3.64)	≤0.01						
ELN (categorical)								
ELN ≥ 16	1.00		1.00		1.00		1.00	
ELN < 16	1.41 (1.23–1.62)	≤0.01	1.08 (0.95–1.24)	0.25	1.04 (0.91–1.19)	0.57	0.59 (0.48–0.71)	≤0.01
LNR category								
LNR1			1.00					
LNR2			1.19 (0.97–1.46)	0.09				
LNR3			1.71 (1.4–2.09)	≤0.01				
LNR4			3.01 (2.47–3.66)	≤0.01				
LODDS category								
LODDS1					1.00			
LODDS2					1.23 (1.01–1.51)	0.04		
LODDS3					1.74 (1.43–2.13)	≤0.01		
LODDS4					3.06 (2.51–3.74)	≤0.01		
NLN category								
NLN1							1.00	
NLN2							1.39 (1.13–1.7)	≤0.01
NLN3							2.61 (2.07–3.29)	≤0.01
NLN4							3.35 (2.6–4.31)	≤0.01

*OS, overall survival; N, number; IQR, interquartile range; HR, hazard ratio; CI, confidence interval; ELNs, Examined lymph nodes; PLN, number of positive lymph nodes; LNR, lymph node ratio; LODDS, log odds of positive lymph node; NLN, negative lymph node.*

**Table 3:** Multivariate Cox regression analysis of prognostic predictors for CSS in gastric adenocarcinoma patients after NAT.

Characteristic	Model1 (ypN)		Model2 (LNR)		Model3 (LODDS)		Model4 (NLN)	
CSS	Hazard ratio (95% CI)	*p*-value	Hazard ratio (95% CI)	*p*-value	Hazard ratio (95% CI)	*p*-value	Hazard ratio (95% CI)	*p*-value
Age								
≤65	1.00		1.00		1.00		1.00	
>65	1.14 (1.00–1.31)	0.06	1.15 (1.00–1.32)	0.04	1.15 (1.00–1.32)	0.05	1.14 (0.99–1.31)	0.06
Histologic type								
Adenocarcinoma	1.00		1.00		1.00		1.00	
Signet ring cell carcinoma	1.26 (1.06–1.49)	≤0.01	1.24 (1.05–1.47)	≤0.01	1.23 (1.04–1.46)	0.02	1.23 (1.04–1.46)	0.02
Grade								
G1/G2	1.00		1.00		1.00		1.00	
G3/G4	1.18 (0.99–1.41)	0.06	1.20 (1.01–1.43)	0.04	1.21 (1.02–1.45)	0.03	1.28 (1.08–1.53)	≤0.01
Unknown	0.98 (0.72–1.34)	0.91	0.99 (0.72–1.35)	0.94	0.99 (0.73–1.36)	0.97	1.08 (0.79–1.47)	0.63
Location								
Upper	1.00		1.00		1.00		1.00	
Middle	0.71 (0.58–0.87)	≤0.01	0.71 (0.58–0.87)	≤0.01	0.71 (0.58–0.87)	≤0.01	0.75 (0.61–0.92)	≤0.01
Lower	0.76 (0.61–0.94)	≤0.01	0.75 (0.60–0.94)	≤0.01	0.75 (0.60–0.94)	0.01	0.77 (0.61–0.96)	0.02
Overlapping	0.95 (0.72–1.25)	0.70	0.95 (0.72–1.25)	0.70	0.95 (0.72–1.25)	0.72	1.01 (0.76–1.33)	0.95
NOS	1.00 (0.72–1.37)	0.98	0.97 (0.71–1.34)	0.87	0.98 (0.71–1.34)	0.88	1.10 (0.80–1.51)	0.56
Tumor size								
≤2 cm	1.00		1.00		1.00		1.00	
>2 cm, ≤5 cm	1.04 (0.82–1.31)	0.75	1.08 (0.86–1.36)	0.51	1.09 (0.86–1.37)	0.48	1.17 (0.93–1.47)	0.19
>5 cm	1.01 (0.80–1.29)	0.91	1.05 (0.82–1.34)	0.70	1.06 (0.83–1.35)	0.63	1.23 (0.97–1.57)	0.08
Linitis plastica	1.13 (0.67–1.9)	0.64	1.13 (0.67–1.89)	0.65	1.14 (0.68–1.91)	0.62	1.05 (0.63–1.77)	0.85
Uknown	0.97 (0.74–1.26)	0.81	1.01 (0.78–1.31)	0.95	1.00 (0.77–1.31)	0.97	1.03 (0.79–1.34)	0.84
ypT category								
ypT1	1.00		1.00		1.00		1.00	
ypT2	1.41 (0.96–2.08)	0.08	1.36 (0.92–2.00)	0.13	1.38 (0.93–2.03)	0.11	1.45 (0.98–2.13)	0.06
ypT3	1.67 (1.19–2.34)	≤0.01	1.58 (1.13–2.21)	≤0.01	1.59 (1.13–2.22)	0.01	1.90 (1.36–2.64)	≤0.01
ypT4a	1.84 (1.31–2.59)	≤0.01	1.75 (1.24–2.46)	≤0.01	1.74 (1.24–2.45)	≤0.01	2.09 (1.49–2.92)	≤0.01
ypT4b	2.12 (1.42–3.17)	≤0.01	2.01 (1.34–3.01)	≤0.01	2.02 (1.35–3.02)	≤0.01	2.49 (1.67–3.70)	≤0.01
ypN category								
ypN0	1.00							
ypN1	1.44 (1.16–1.77)	≤0.01						
ypN2	1.99 (1.61–2.45)	≤0.01						
ypN3	3.20 (2.56–4.01)	≤0.01						
ELN (categorical)								
ELN ≥ 16	1.00		1.00		1.00		1.00	
ELN < 16	1.45 (1.26–1.67)	≤0.01	1.09 (0.95–1.26)	0.22	1.04 (0.90–1.20)	0.57	0.58 (0.48–0.71)	≤0.01
LNR category								
LNR1			1.00					
LNR2			1.26 (1.01–1.57)	0.04				
LNR3			1.85 (1.50–2.29)	≤0.01				
LNR4			3.19 (2.59–3.94)	≤0.01				
LODDS category								
LODDS1					1.00			
LODDS2					1.34 (1.08–1.66)	≤0.01		
LODDS3					1.91 (1.53–2.36)	≤0.01		
LODDS4					3.30 (2.66–4.10)	≤0.01		
NLN category								
NLN1							1.00	
NLN2							1.39 (1.12–1.73)	≤0.01
NLN3							2.73 (2.13–3.48)	≤0.01
NLN4							3.44 (2.64–4.49)	≤0.01

*CSS, cancer specific survival; N, number; IQR, interquartile range; HR, hazard ratio; CI, confidence interval; ELN, Examined lymph nodes; PLN, number of positive lymph nodes; LNR, lymph node ratio; LODDS, log odds of positive lymph node; NLN, negative lymph node.*

### Survival Analysis

**Figure 2:**
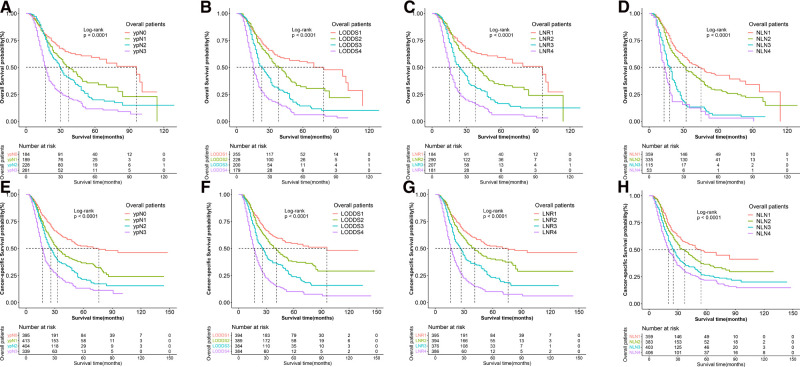
Kaplan–Meier survival curves of OS according to (**A**) N stage, (**B**) LODDS, (**C**) LNR, and (**D**) NLN staging systems and CSS according to (**E**) N stage, (**F**) LODDS, (**G**) LNR and (**H**) NLN staging system for gastric adenocarcinoma patients after NAT. OS, overall survival; CSS, cancer specific survival; LNR, lymph node ratio; LODDS, log odds of positive lymph node; NLN, negative lymph node; NAT, neoadjuvant therapy.

The median CSS and OS of patients were 29 and 27 months, and death caused by gastric adenocarcinoma was observed in 872 (56.2%) patients while 975 (62.9%) patients died from any causes. As shown in Table 1 and Figure 2, four lymph node staging schemes were correlated to CSS and OS and pairwise survival in different schemes between subgroups was statistically significant.

The 3-year CSS and OS rates in the ypN0 group (*n* = 395), ypN1 group (*n* = 413), ypN2 group (*n* = 404) and ypN3 group (*n* = 339) were 55.8%, 36.7%, 21.5%, 13.3% and 46.6%, 31.7%, 18.9%, 10.3%, respectively. The 3-year CSS and OS rates in the LNR1 group (*n* = 395), LNR 2 group (*n* = 394), LNR 3 group (*n* = 376) and LNR 4 group (*n* = 386) were 52.3%, 40.3%, 23.3%, 10.2% and 46.0%, 35.6%, 20.4%, 7.4% respectively. The 3-year CSS and OS rates in the LOODS1 group (*n* = 394), LOODS2 group (*n* = 389), LOODS3 group (*n* = 384) and LOODS4 group (*n* = 384) were 54.8%, 38.0%, 24.0%, 10.3% and 48.5%, 33.5%, 20.9%, 7.4%, respectively. The 3-year CSS and OS rates in the NLN1 group (*n* = 359), NLN 2 group (*n* = 383), NLN 3 group (*n* = 403) and NLN 4 group (*n* = 406) were 47.6%, 36.6%, 26.2%, 21.9% and 42.8%, 31.0%, 22.7%, 18.3%, respectively.

### Prognostic Performance of Four Lymph Node Models

**Figure 3:**
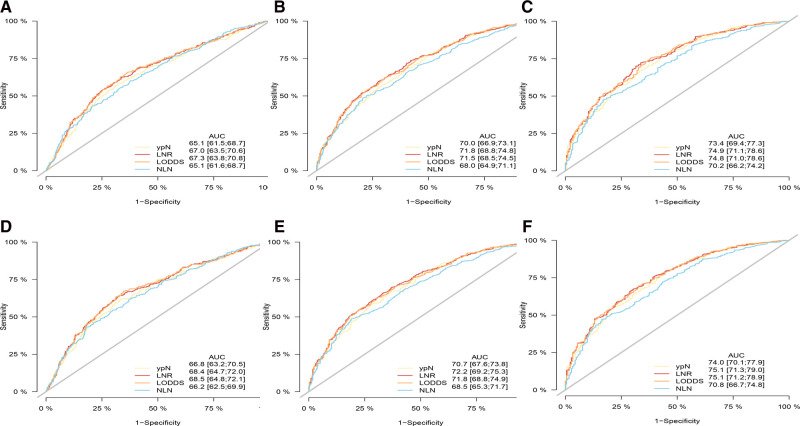
ROC curve of the ypN stage, LNR, LODDS and NLN in the prediction of prognosis of gastric adenocarcinoma patients after NAT at 1 (**A**), 3 (**B**), 5 (**C**) year point for OS and 1 (**D**), 3 (**E**), 5 (**F**) year point for CSS when LN status assessed as categorical varaible. OS, overall survival; CSS, cancer specific survival; ROC, receiver operative curve; LNR, lymph node ratio; LODDS, log odds of positive lymph node; NLN, negative lymph node.

**Figure 4:**
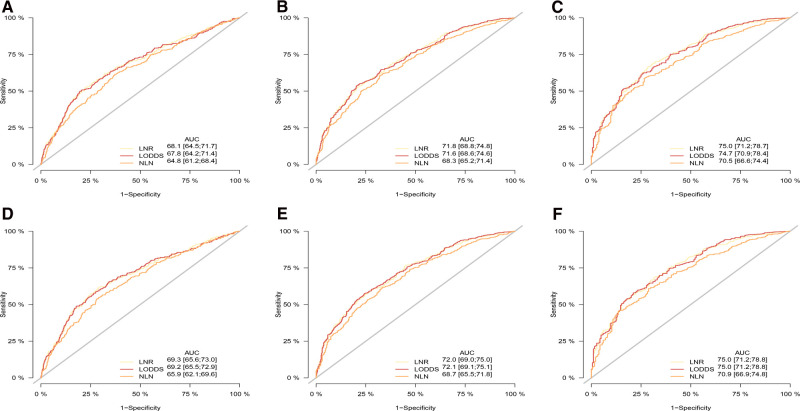
ROC curve of the ypN stage, LNR, LODD and NLN in the prediction of prognosis of gastric adenocarcinoma patients after NAT at 1 (**A**), 3 (**B**), 5 (**C**) year point for OS and 1 (**D**), 3 (**E**), 5 (**F**) year point for CSS when LN status assessed as continuous varaible, OS, overall survival; CSS, cancer specific survival; ROC, receiver operative curve; LNR, lymph node ratio; LODDS, log odds of positive lymph node; NLN, negative lymph node.

As shown in Tables 4, 5, when regarded as the categorical variables, LNR and LODDS had better performance than ypN and NLN schemes with higher C-index, lower AIC, higher LR test. Interestingly, when assessed as the continuous variables, the predictive performance of all staging systems were improved and LODDS outperformed other staging systems for CSS with higher C-index (CSS: 0.665), lower AIC (CSS: 11,422.83), higher LR test (CSS: 278.6) as well as LNR had better performance for OS with C-index (0.661), AIC (12,720.98), LR test (283.2). Additionally, as shown in Figure 3, based on the analysis of ROC curves, when regarded as the categorical variable, LNR and LODDS showed the superior discriminatory power for OS (LNR: 5-year CSS:0.751, 5-year OS:0.749;)(LODDS: 5-year CSS:0.751, 5-year OS:0.748;)to ypN (categorical:5-year CSS:0.740, 5-yearvOS:0.739;), NLN (categorical: 5-year CSS:0.708, 5-year OS: 0.702;) and the same result appeared when referring to CSS. The similar findings persisted when values of schemes were analyzed as the continuous variables (Figure 4).

**Table 4:** Predictive performance of different staging systems in subgroups when LN status assessed as categorical varaible.

Study cohort (categorical)	Overall			ELNs < 16			ELNs ≥ 16			N0		
	AIC	C-index (95% CI)	LR test	AIC	C-index (95% CI)	LR test	AIC	C-index (95% CI)	LR test	AIC	C-index (95% CI)	LR test
CSS												
Model 1 (ypN)	11,472.87	0.653 (0.624–0.664)	232.6	4,858.80	0.651 (0.602–0.658)	102.1	5,435.35	0.662 (0.616–0.672)	130.4			
Model 2 (LNR)	11,445.35	0.661 (0.633–0.672)	260.1	4,848.91	0.654 (0.603–0.660)	112.1	5,408.93	0.677 (0.635–0.688)	156.8			
Model 3 (LODDS)	11,448.15	0.661 (0.632–0.671)	257.3	4,851.36	0.654 (0.602–0.659)	109.5	5,411.16	0.675 (0.634–0.686)	154.6	1,675.01	0.667 (0.582–0.669)	45.80
Model 4 (NLN)	11,508.48	0.643 (0.613–0.653)	197.3	4,891.65	0.627 (0.578–0.638)	67.23	5,432.53	0.659 (0.611–0.664)	133.2	1,671.14	0.675 (0.587–0.674)	51.68
OS												
Model 1 (ypN)	12,781.56	0.645 (0.616–0.655)	226.6	5,406.97	0.640 (0.593–0.647)	95.04	6,048.28	0.654 (0.610–0.662)	131.1			
Model 2 (LNR)	12,745.24	0.656 (0.629–0.666)	262.9	5,391.14	0.649 (0.603–0.657)	110.9	6,019.71	0.669 (0.629–0.680)	159.7			
Model 3 (LODDS)	12,748.61	0.656 (0.629–0.666)	259.5	5,393.35	0.649 (0.602–0.656)	108.7	6,020.50	0.669 (0.628–0.679)	158.9	2,012.14	0.664 (0.589–0.669)	56.34
Model 4 (NLN)	12,812.02	0.638 (0.609–0.646)	196.1	5,435.69	0.621 (0.571–0.626)	64.33	6,043.23	0.653 (0.610–0.661)	136.2	2,007.88	0.672 (0.596–0.675)	62.60

*ELN, Examined lymph nodes;LNR, lymph node ratio; LODDS, log odds of positive lymph node; NLN, negative lymph node; C-index, Harrell’s concordance index; ROC, the Receiver Operative Curve; LR test, the likelihood ratio test; AIC, the Akaike information criterion.*

**Table 5:** Predictive performance of different staging systems in subgroups when LN status assessed as continuous varaible.

Study cohort (continuous)	Overall			ELNs < 16			ELNs ≥ 16			N0		
	AIC	C-index (95% CI)	LR test	AIC	C-index (95% CI)	LR test	AIC	C-index (95% CI)	LR test	AIC	C-index (95% CI)	LR test
CSS												
Model 1 (ypN)	11,474.82	0.652 (0.625–0.663)	226.7	4,853.54	0.651 (0.605–0.661)	103.3	5,426.91	0.664 (0.623–0.6745)	134.8			
Model 2 (LNR)	11,428.77	0.665 (0.638–0.677)	272.7	4,835.69	0.659 (0.613–0.670)	121.2	5,399.79	0.678 (0.637–0.689)	161.9			
Model 3 (LODDS)	11,422.83	0.665 (0.639–0.678)	278.6	4,841.13	0.658 (0.612–0.669)	115.8	5,392.99	0.677 (0.635–0.690)	168.8	1,670.67	0.670 (0.592–0.679)	48.15
Model 4 (NLN)	11,511.07	0.643 (0.615–0.657)	190.4	4,876.43	0.636 (0.589–0.648)	80.4	5,449.78	0.652 (0.611–0.667)	112.00	1,666.38	0.673 (0.594–0.680)	52.43
OS												
Model 1 (ypN)	12,776.81	0.647 (0.622–0.658)	227.3	5,400.24	0.642 (0.598–0.652)	97.8	6,033.89	0.660 (0.620–0.671)	141.5			
Model 2 (LNR)	12,720.98	0.661 (0.636–0.673)	283.2	5,374.12	0.655 (0.610–0.665)	123.9	6,007.54	0.671 (0.631–0.683)	167.8			
Model 3 (LODDS)	12,723.34	0.660 (0.634–0.672)	280.8	5,384.00	0.653 (0.610–0.664)	114.0	6,003.26	0.668 (0.630–0.684)	172.1	2,009.66	0.667 (0.595–0.675)	56.82
Model 4 (NLN)	12,816.52	0.638 (0.611–0.651)	187.6	5,419.04	0.632 (0.587–0.643)	79.0	6,062.16	0.646 (0.607–0.659)	113.2	2,005.81	0.670 (0.599–0.678)	60.67

*ELN, Examined lymph nodes;LNR, lymph node ratio; LODDS, log odds of positive lymph node; NLN, negative lymph node; C-index, Harrell’s concordance index; ROC, the Receiver Operative Curve; LR test, the likelihood ratio test; AIC, the Akaike information criterion.*

### Prognostic Performance of Lymph Node Models in the Subgroups of <16 ELN and ≥16 ELN

**Figure 5:**
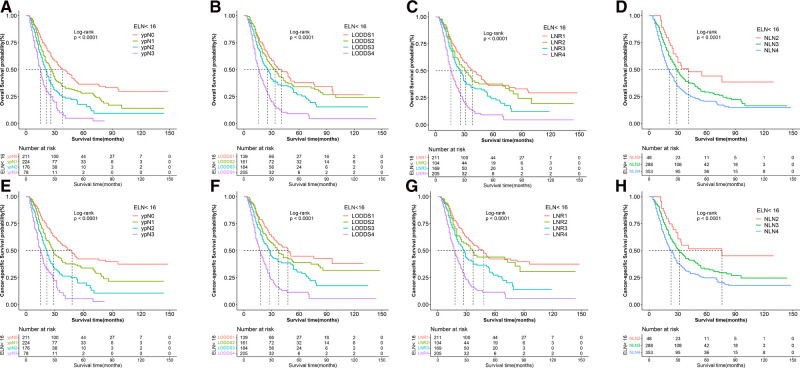
Kaplan–Meier survival curves of OS according to (**A**) ypN (**B**) LODDS, (**C**) LNR, and (**D**) NLN staging system and CSS according to (**E**) ypN, (**F**) LODDS, (**G**) LNR and (**H**) NLN staging system for gastric adenocarcinoma patients after NAT with ELN < 16. OS, overall survival; CSS, cancer specific survival; LNR, lymph node ratio; LODDS, log odds of positive lymph node; NLN, negative lymph node; NAT, neoadjuvant therapy.

We did a subgroup analysis based on the ELN for patients with insufficient ELN. When seen as the categorical variables, pairwise survival in ypN and NLN staging between subgroups was statistically significant. However, as shown in Figure 5, no significant differences were found between the LNR1 and LNR2 groups (OS: *p* = 0.26, CSS: *p* = 0.28), the LNR2 and LNR3 groups (OS: *p* = 0.07), or the LODDS1 and LODDS2 groups (OS: *p* = 0.34, CSS: *p* = 0.21). As shown in Table 4, LNR had better discrimination power (CSS: 0.654, OS: 0.649), higher homogeneity (CSS: 112.1, OS: 110.9), and better model fitness (CSS: 4,848.91, OS: 5,391.14) for CSS and OS compared to other staging systems. When lymph node staging systems were viewed as continuous variables shown in Table 5, a similar result was obtained.

**Figure 6:**
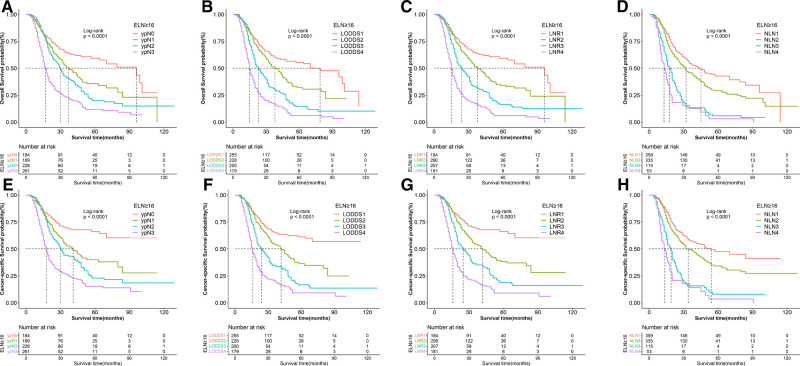
Kaplan–Meier survival curves of OS according to (**A**) ypN, (**B**) LODDS, (**C**) LNR, and (**D**) NLN staging system and CSS according to (**E**) ypN, (**F**) LODDS, (**G**) LNR and (**H**) NLN staging system for gastric adenocarcinoma patients after NAT with ELN ≥ 16. OS, overall survival; CSS, cancer specific survival; LNR, lymph node ratio; LODDS, log odds of positive lymph node; NLN, negative lymph node; NAT, neoadjuvant therapy.

For patients with adequate ELN, when assessed as the categorical variable, as shown in Figure 6, pairwise survival in LNR, LOODS, and NLN staging between subgroups was statistically significant. However, there was no significant difference between the ypN1 and ypN2 groups (OS: *p* = 0.06). Furthermore, As shown in Table 4, LNR had better prognostic performance than others, with a higher C-index (CSS: 0.677, OS: 0.669), lower AIC (CSS: 5,408.93, OS: 6,019.71) and higher LR test (CSS: 156.8, OS: 159.7). When viewed as a continuous variable shown in Table 5, LNR had greater discriminatory power (CSS: 0.678, OS: 0.671). In comparison, LODDS had a lower AIC (CSS: 5,392.99, OS: 6,003.26) and a higher LR test (CSS: 168.8, OS: 172.1).

### Prognostic Performance of Lymph Node Models in the ypN0 Subgroup

**Figure 7:**
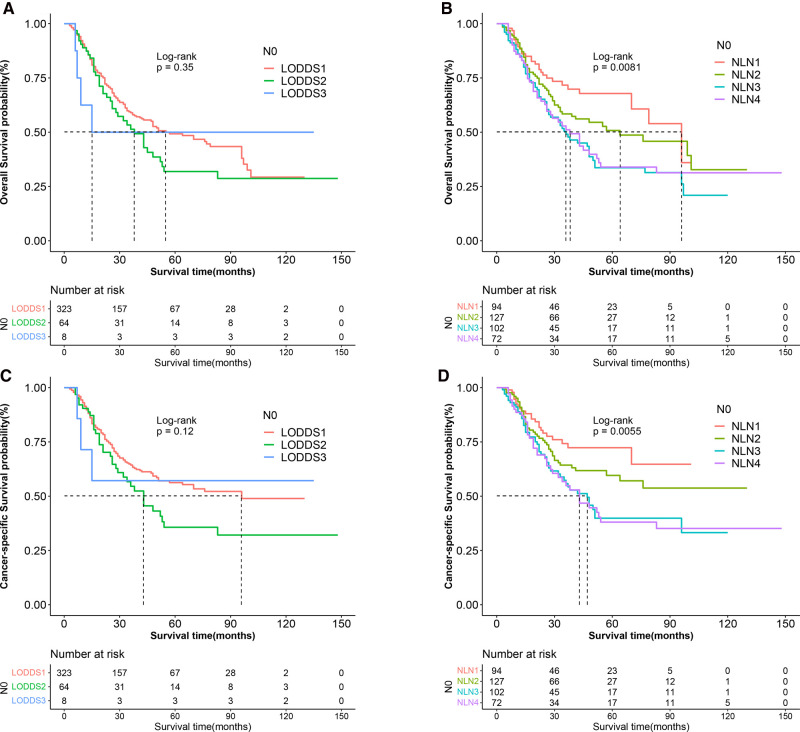
Kaplan–Meier survival curves of OS according to (**A**) LOODS, (**B**) NLN staging system and CSS according to (**C**) LODDS, and (**D**) NLN staging system for gastric adenocarcinoma patients after NAT with ypN0. (OS, overall survival; CSS, cancer specific survival; LODDS, log odds of positive lymph node; NLN, negative lymph node; NAT, neoadjuvant therapy.

Lymph node downstaging is common among patients treated with NAT, resulting in an increasement in the frequency of node-negative patients and patients with inadequate ELN. However, as shown in Figure 7, LNR and ypN staging systems demonstrated no predictive value in node-negative patients, whereas LODDS and NLN could still be utilized to stratify patients. As demonstrated in Tables 4, 5, regardless of whether the variable is considered categorical or continuous in nature, NLN showed superior predictive performance for CSS and OS. Given the simplicity with which NLN may be calculated, it seems that NLN might be a feasible option for evaluating prognosis in ypN0 patients.

### Distributive Correlation Between LODDS, LNR, and NLN

**Figure 8:**
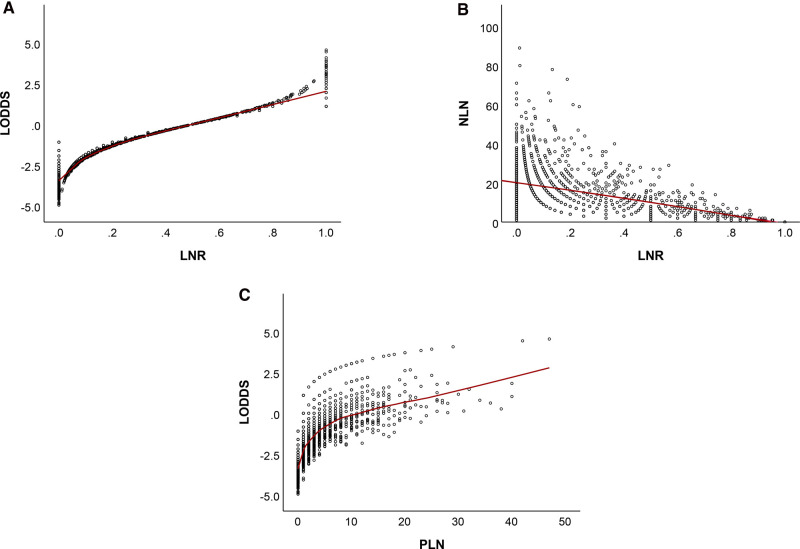
Distribution of LNR vs LODDS (**A**), NLN (**B**), and PLN vs LODDS (**C**). LNR, lymph node ratio; LODDS, log odds of positive lymph node; NLN, negative lymph node; PLN, number of positive lymph nodes.

Figure 8 showed that LNR has a substantial correlation with LODDS, NLN. Furthermore, there was still heterogeneity in survival in patients with node-negatvie or no negative lymph node, which was well stratified by the NLN and LODDS when compared to the ypN and LNR staging schemes. As LNR increased, LODDS increased nonlinearly. The correlation is heterogeneous when the LNR approaches extreme levels, demonstrating that the NLN and LODDS systems might differentiate survival in patients with extreme LNR scores.

## Discussion

At present, NCCN guidelines recommend at least 16 lymph nodes should be harvested to decrease stage migration regardless of the initial treatment. However, following neoadjuvant therapy, there is a decrease in ELN and PLN as a result of stromal atrophy, fibrosis, and lymph node shrinking [7, 32–34]. It was demonstrated that lymph node status after NAT was still an important predictor of prognosis and that adequate N stage assessment may aid in post-operative treatment decision [10–12]. Thus, relying exclusively on the ypN stage to determine LN status may understage patients, thereby influencing postoperative treatment decision-making and prognosis. As a result, we sought to determine the optimal staging system among ypN, LNR, LODDS, and NLN in assisting in post-operative care of patients with gastric adenocarcinoma following NAT.

Currently, the eighth edition of the AJCC N staging has been criticized for requiring sufficient lymph node resection to minimize staging migration. In the past, some researchers proposed the optimal number of examined lymph nodes and several alternative methods of calculating the lymph node status. However, the majority of these studies were conducted on patients who have not had preoperative therapy, and their applicability to patients who have received preoperative treatment has not been established. The current study is one of the biggest database analyses yet conducted to evaluate the four staging systems used in gastric adenocarcinoma following preoperative therapy, as well as the first report to evaluate the prognostic power of four staging systems among patients with less extended lymph node dissection (less than 16 lymph nodes harvested) following NAT. It was discovered that four lymph node staging schemes were capable of stratifying patients for CSS and OS, and that all four were significant independent prognostic variables in multivariate analysis. LNR and LODDS outperformed other alternative staging systems regardless of whether the variable was categorical or continuous in nature. It’s worth noting that the NLN staging system outperformed others among node-negative patients.

It was demonstrated that LNR and LODDS integrated the ELN and PLN to minimize stage migration in a variety of cancers, and both outperformed the N stage for prognosis prediction [8, 13, 15, 16, 18]. Similarly, when the LNR and LOODS staging systems were applied in gastric adenocarcinoma following neoadjuvant treatment, similar conclusions were observed. However, in reviewing the literature, which of the two systems has the superior predictive ability remains controversial. Smith, Nelson, and Schwarz et al. validated the finding that LNR was the best staging scheme for predicting prognosis in patients with resectable gastric adenocarcinoma when used as a categorical variable [35], whereas Wang et al found that LODDS showed a prognostic superiority over both pN and LNR staging systems, when assessed as categorical variable [36]. Interestingly, in a multi-institutional database study, Spolverato et al found that LNR assess LN status with great predictive power when assessed as the categorical variable, while LOODS became the better predictive factor when regarded as the continuos variable for gastric adenocarcinoma patients without neoadjuvant therapy [17]. In the current study, similar findings were observed that LNR had better predictive ability than LODDS when regarded as the categorical variable, whereas LOODS became the better predictive factor for CSS when regarded as the continuos variable. A possible explanation for this might be that when stratifying patients based on LN status, numerous alternative “optimal” cutoffs have been suggested for each system. For instance, Marchet et al. proposed the four-category cutoff for LNR as 0–10%–25%, whereas Sun et al selected 0–20%–50%. In addition, Wang et al advocated the five-category cutoff based on LNR. However, the factors related to the cutoff selection may include the many aspects including the different statistical methods, the invloved patients selected form different countries, and patients following different preoperative treatments and so on [8, 17, 37].

When further exploited the relationship between the two staging systems, in patients reaching extremely low or high LNR scores, LNR staging system failed to stratify the prognosis. When seen in the scatter plot (Figure 8), as LNR was increased, LODDS nonlinearly increased. However, when the LNR approaches extreme LNR levels, the connection between the two variables becomes heterogeneous, showing that the LODDS system is capable of discriminating between patients with varying survival status due to their high LNR score. These results may seem self-evident. However, most doctors would agree that patients with one ELN and one PLN have a different prognosis when compared with those with twelve ELN and twelve PLN.

In our multivariate Cox regression analysis, ELN was an independent prognostic factor, suggesting that checking more than 16 lymph nodes can achieve better survival and help predict the prognosis of patients accurately. Patients with insufficient lymph node harvest accounted for 44.4 percent of total patients in our research, indicating the outcome of preoperative therapy. Hence, we performed subgroup analysis based on the ELN. When assessed as the categorical variable, LNR had the better prognostic performance than others regardless of the status of ELN. When the lymph node staging systems were regarded as continuous variable, LNR persisted to have better discrimination ability for OS. However, this result has not previously been described. Yang et al found that LODDS showed better predictive performance than pN, LNR, and NLN among adenocarcinoma of esophagogastric junction patients with ≤16 ELN [38]. Xu et al proposed that LODDS was a better prognostic factor for DFS than ypN staging or the LNR-based system in patients with rectal cancer after NAT, particularly in patients with insufficent ELN [8]. In a retrospective study, Spolverato et al found LOODS outperformed LNR when less than 10 lymph node harvested in gastric cancer with cancer-directed sugery [17]. Some researchers previously stated that one of the limitations of any LN ratio scheme is an inadequate number of LNs, and emphasized that utilizing an LN ratio scheme does not effectively compensate for low LN counts [37, 39, 40]. This discrepancy may be explained by the fact that preoperative therapy increased the frequency of node-negative patients who serve as respond a critical indicator of the efficacy of preoperative treatment. In a retrospective study including 316 patients with gastric cancer following NAT, Ikoma et al found similar survival in patients achieveing ypN0 regardless of the clinical N stage, suggesting that ypN0 status is an important hallmark demonstrating the effectiveness of preoperative therapy. Therefore, the homogeneity and model fit of LNR staging system has been improved within LNR1 group. There was the evidence that LNR had better discriminating capacity and homogeneity than LODDS in our investigation.

Indeed, in node-negative patients, ELN has been shown to be a indepent prognostic factor and is equal to NLN [9]. LODDS is different from ELN but still reply on ELN. In our study, ELN was an independent prognostic factor with CSS and OS in multivariate Cox regression analysis. Analyed by Kaplan-Meier method and Log-rank test, no statistically significant difference were observed between LODDS subgroups while there was significantly different in NLN (NLN1 vs NLN4, P < 0.05;NLN2 vs NLN4, P< 0.05). Furthermore, NLN outperformed LODDS with better discrimination power, higher homogeneity and better model fitness for CSS and OS. Considering the user-friendly calculation of NLN, it should be recommended in clinical prognostic assessment as a substitute for assessing lymph node status in ypN0 patients.

Even though the three alternative staging schemes (LNR,LODDS,and NLN) outperformed the ypN stage in different aspects, it is currently not widely applied in clinical practice. There could be several explanations for this, including the following: To begin, unlike the ypN stage, consensus on the cut-off value for alternative systems remains elusive. Secondly, when referring to LOODS, the calculation is not user-friendly and inconvenient. Finally, except for patients at high risk of N stage migration, it failed to provide a significant improvement in predicted prognosis.

For the reasons stated above, when LN status were seen as continuous factors rather than categorical variables, the predictive performance of three staging systems (LNR, LODDS, and NLN) was enhanced. Spolverato et al. concluded in a similar manner that staging methods should evaluate LN status as continuous variables rather than using indiscriminate categorical cutoffs for patients with gastric adenocarcinoma undergoing cancer-directed surgery [17]. Thus, our findings implies that in gastric adenocarcinoma following NAT, the staging system should consider LN status as a continuous variable rather than a heterogeneous categorical variable.

This study has some limitations. To begin, unavoidable deviations occur as a result of the retrospective experimental design. To ensure the reliability of the results, they must be validated in large, multi-center, prospective clinical trials. Second, the SEER database’s lack of comprehensive data (such as surgical margin status, etc.) precludes future in-depth research. Finally, because our study excluded patients in stage IV, the impact on these patients remains unknown.

## Conclusions

LNR had a better predictive performance than ypN, LODDS and NLN staging systems regardless of the status of ELN when regarded as the categorical variable, whereas LOODS became the better predictive factor for CSS when regarded as the continuos variable. In node-negative patients, NLN might be a feasible option for evaluating prognosis and a combination of LNR and NLN should be considered as user-friendly method in the clinical prognostic assessment.

## Data Availability

The datasets presented in this study can be found in online repositories. The names of the repository/repositories and accession number(s) can be found in the article.
